# Experimental Investigation of the Effect of Manufactured Sand and Lightweight Sand on the Properties of Fresh and Hardened Self-Compacting Lightweight Concretes

**DOI:** 10.3390/ma9090735

**Published:** 2016-08-29

**Authors:** Yiyun Zhu, Hongzhi Cui, Waiching Tang

**Affiliations:** 1 School of Civil Engineering and Architecture, Xi’an University of Technology, Xi’an 710048, China; zyyun@xaut.edu.cn; 2 Guangdong Provincial Key Laboratory of Durability for Marine Civil Engineering, College of Civil Engineering, Shenzhen University, Shenzhen 518060, China; 3 School of Architecture and Built Environment, The University of Newcastle, Callaghan, NSW 2308, Australia; patrick.tang@newcastle.edu.au

**Keywords:** self-compacting lightweight concrete, manufactured sand, lightweight sand, sand ratio, flowability, mechanical properties, water permeability coefficient

## Abstract

Self-compacting lightweight concrete (SCLC) is a promising construction material for building applications, but most SCLCs today are made with river sand (RS). There is an increasing demand for environmental protection, as well as materials with a high strength/density ratio. The manufactured sand (MS) and lightweight sand (LS) as fine aggregates in cement-based composite materials have been receiving more attention among researchers. However, there is not much information about the effects of MS and LS on the properties of the fresh and hardened SCLCs. In this paper, the properties of fresh and hardened SCLC made with MS and LS were investigated by a series of experiments. SCLCs made with RS served as the control in this study. The test results show that increasing the sand ratio (from 0.40–0.50) decreased the filling ability and led to an increased T_50_ time, which is the time spent for the concrete to reach the 500 mm spread circle, for all of the fresh SCLCs. Although the passing ability of MS-SCLCs and LS-SCLCs is not as good as RS-SCLCs, their results are still within an acceptable range. The ratio of mechanical properties to density was found to increase with an increase of the sand ratio for all of the hardened SCLCs. MS-SCLCs presented the highest compressive strength among all of the SCLCs studied. Although the mean compressive strength of LS-SCLCs is lower than those of the other two SCLCs by 8%, their strength to density ratio is higher than others by 15%, and the ratio increases remarkably with the increase of the sand ratio. Permeability test results showed that the permeability coefficient of MS-SCLC is remarkably lower than that of LS-SCLC, but slightly higher than that of RS-SCLC.

## 1. Introduction

Lightweight concrete (LWC) is an attractive building material for its competitive high strength/density ratio. Therefore, there is a growing interest among engineers to use LWCs, including lightweight aggregate concretes and polymer concretes [1], for structural applications. On the other hand, self-compacting concrete (SCC) is able to flow under its own weight and fills formwork completely without the requirement for mechanical compaction [2,3,4]. It is generally believed that SCC is a noise-free environmentally-friendly cement-based material [5]. Self-compacting lightweight concrete (SCLC), which combines the advantages of both SCC and LWC, is one of the promising construction materials for building applications. However, most SCLCs nowadays are made with river sand (RS). In China, RS is the most widely-employed fine aggregate for concrete production, but the overexploitation of RS to meet the demand of the application has resulted in many harmful consequences, such as increasing depths of river beds, lowering water levels and salinity intrusion into rivers. Due to these environmental issues, the Chinese government has imposed various restrictions on the extraction of RS, particularly in vulnerable locations. Meanwhile, concrete suppliers are encouraged to use a number of RS substitutes, such as manufactured sand (MS), dredged marine sand (DMS), lightweight sand (LS), etc., to produce concrete.

In the past, researchers have studied the workability of fresh SCLC and the mechanical properties of SCLC made with RS [6,7,8]. However, the studies of different fine aggregates on the properties of fresh and hardened SCLC are limited. Topçu et al. [9] studied the effect of aggregate types on properties of hardened SCLC. Three types of coarse lightweight aggregate (LWA), namely pumice, volcanic tuff and diatomite, and normal limestone aggregate were used. The SCLC mixtures were prepared with different combinations of the water to binder ratio and superplasticizer dosage. Their results, in general, showed that SCLC with LWA has lower mechanical and physical properties, except for thermal properties, when compared to the properties of SCC. Bouziani [10] investigated the fresh properties and compressive strength of self-compacting concrete made with different sand types (river sand, crushed sand and dune sand) by a mixture design modelling approach. It has been reported that different fine aggregates may have different micro-particle addition effects on the internal dissipation of concrete. Sobuz et al. [11] studied the influence of a wide range of fine aggregate sources and grading on the workability of ultra-high performance concrete by utilizing four fine aggregates (a washed river sand, a mined sand, a manufactured sand and a granulated lead smelter slag). The fineness modulus of these four fine aggregates is different and ranges from 2.13–4.01. Their results show that using conventional natural and manufactured fine aggregates, including granulated slag, it is possible to produce concrete with good workability and compressive strengths in the range of 130–160 MPa without the need for complex mixing or curing regimes.

Although research on different RS replacements, such as manufactured sand (RS) [12], seashells [13], rich husk ash [14], etc., serving as fine aggregates of SCC has been carried out, these studies did not provide a comparison between different fine aggregates. In the past, the majority of research was focused on general SCC; nowadays, however, more and more attention has been paid to SCLC. Moreover, the effects of fine aggregate on the mechanical and durability properties of SCLC are still uncertain and yet to be determined. The inherent high porosity of the lightweight aggregates, the likelihood of the high permeability of lightweight concrete and whether or not SCLC has a good durability performance in the long term are still questionable.

The aim of this research is to study the effect of manufactured sand (MS) and lightweight sand (LS) on the properties of fresh and hardened SCLC. SCLCs with target strengths ranging from 35–45 MPa and having bulk densities less than 1800 kg/m^3^ were studied. Moreover, the effect of sand ratios on the performance of fresh and hardened SCLC was also studied. Although previous studies published [15,16,17,18] on solid skeleton packing density have explained theoretically how the fine aggregate properties effect the workability of self-compacting concrete, most of these theoretical analyses are usually qualitative in nature, and it is difficult to give a substantial conclusion of how MS and LS would quantitatively influence the fresh and hardened SCLC. It is believed that the results of this experimental investigation can be used to support engineering applications of SCLC made with MS and LS.

## 2. Materials and Methods

### 2.1. Materials and SCLC Mix Proportions

Ordinary Portland cement (OPC), meeting the requirements of BS EN 197-1:2001, with a 28-day mortar compressive strength of 57 MPa, was employed for preparing the SCLC. The specific gravity of cement was 3.4 g/cm^3^. Its initial setting time was 2 h and 35 min, and the final setting time was 8 h 15 min.

**Figure 1:**
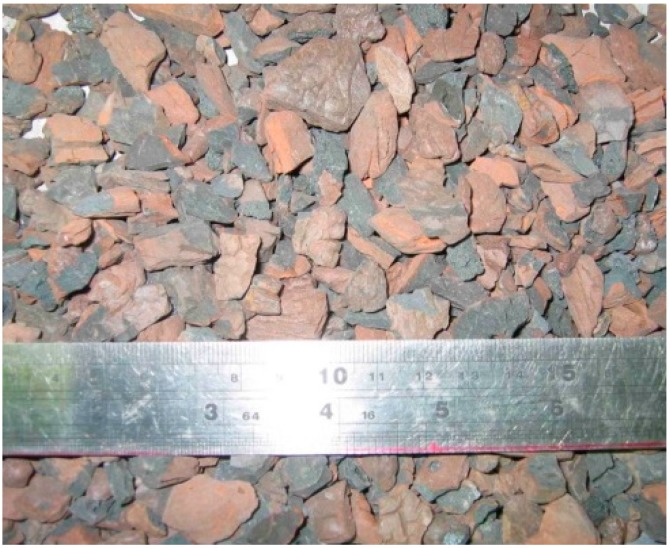
Expanded shale lightweight aggregate.

**Figure 2:**
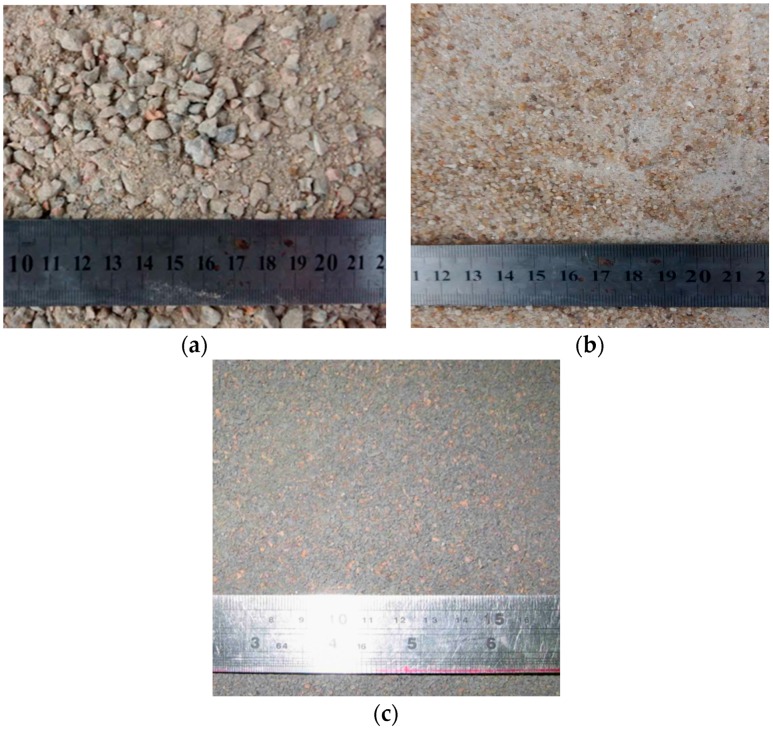
Appearances of the fine aggregates. (**a**) River sand; (**b**) manufactured sand; (**c**) lightweight sand.

There are different methods of making lightweight concretes [19]. In this study, the coarse aggregate used for SCLC was expanded shale lightweight aggregate (LWA). Figure 1 and Table 1 display the appearance and the properties of the LWA, respectively. The river sand (RS), manufactured sand (MS) of granite and lightweight sand (LS) of crushed expanded shale were used as fine aggregates in this study. RS served as the control fine aggregate. The appearances of these fine aggregates are displayed in Figure 2. From Figure 2, it can be shown that the particle shape of RS is nearly spherical. In contrast, the particle shape of MS and LS is angular. Table 2 lists the fineness modulus and grading of the different fine aggregates. In general, MS is coarser than LS and RS. LS is uniformly graded with a 2.36-mm maximum size. As indicated in Table 2, the fineness modulus of RS, MS and LS is 2.77, 3.45 and 2.96, respectively.

A locally-available superplasticizer that complied with BS EN 480-13:2009 was utilized to achieve the expected workability in the SCLC mixes. To minimize segregation in SCLC, researchers used additives and fillers to enhance the viscosity of the mixes of cement-based materials [20,21,22,23]. The effect of different fillers on the properties of cement-based composite can be found from the previous study [24]. An agent for modifying viscosity was utilized to increase the segregation resistance of SCLC. Pulverized fuel ash (PFA) that complied with BS 3892 was also added into the SCLC mix as a mineral filler to prevent the separation of coarse particles in SCLC.

Table 3 shows the details of the mix proportion for SCLCs with a water/binder ratio of 0.40. In this study, the binder content in all mixes is 550 kg/m^3^. To study the influence of sand ratio on SCLC, three sand ratios of 0.40, 0.45 and 0.50 were used. The sand ratio is the percentage of sand in total aggregate by weight. Pulverized fuel ash was used to replace 40% by mass of Portland cement. Due to their high absorption characteristics, both LWA and LS were pre-wetted before mixing the concretes. For manufacturing lightweight aggregate concrete, there has been a mature pre-wetted method in the concrete industry. The pre-wetted method in this research is the same as that of the authors’ previous research [25,26,27].

### 2.2. Materials and Mix Proportions

#### 2.2.1. Fresh Concrete Tests

All of the preparation methods for the SCLCs were in accordance with EN 206-9:2010 (Specification and Guidelines for Self-Compacting Concrete). The slump flow test and the L-box test that complied with EN 12350-8:2010 and EN 12350-10:2010, respectively, were carried out to assess the properties of the fresh concrete. These two tests evaluate the primary characteristics of fresh SCLC and were carried out directly after concrete mixing.

**Figure 3:**
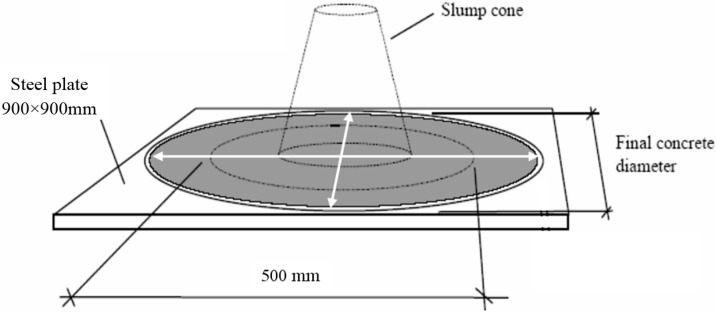
Schematic drawing of the slump flow test.

The slump flow test evaluates the flowability of SCC without any obstruction. The diameter of the slump flow (Figure 3) is the measurement required for all SCLC. EN 12350-8:2010 states that self-compacting concrete should have a slump flow value of between 550 mm and 850 mm, while T_50_ time are the time spent for the concrete to reach the 500 mm spread circle (see Figure 3). The T_50_ times less than two seconds result in a VS1 classification (viscosity classes expressed by T_50_), while T_50_ times greater than or equal to two seconds result in a VS2 classification.

**Figure 4:**
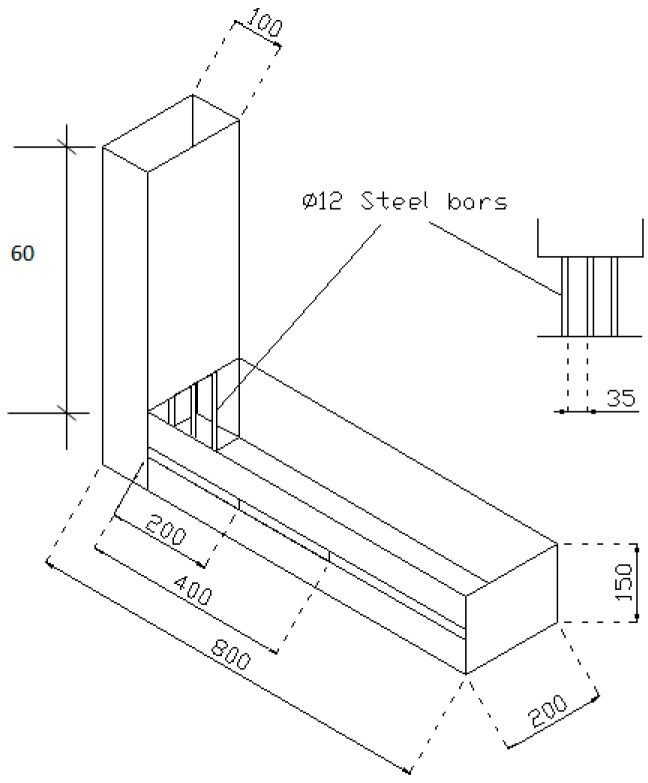
Schematic drawing of the L-box test.

The L-box test (see Figure 4) was performed in accordance with EN 12350-10:2010.

#### 2.2.2. Hardened Concrete Tests

**Figure 5:**
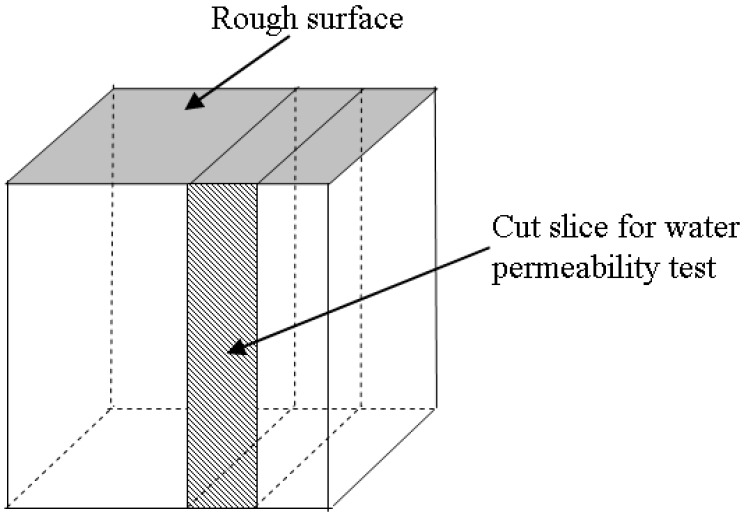
Schematic drawing for the cut slice sample for the water permeability test.

**Figure 6:**
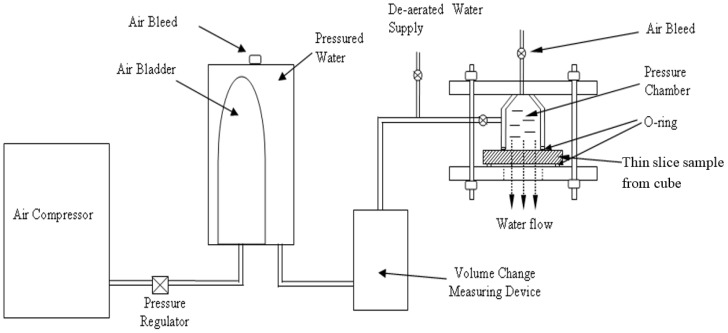
Schematic drawing of the uniaxial water permeability test.

Samples with different sizes were used to investigate the various parameters. One-hundred millimetre cubes were used for the compressive strength test. The elastic modulus test was carried out by cylinders of 100 mm (diameter) × 200 mm (length). Slices of 100 mm × 100 mm × 20 mm in size were used for water permeability tests. The samples were demoulded in about 24 h after casting and then cured in water at 20 ± 3 °C. Samples were taken out for compressive strength, elastic modulus and water permeability tests after 28 days at 20 ± 3 °C of water curing. In each compressive strength test, three cube samples were used. For the modulus of elasticity and water permeability tests, 2 cylinders and 2 prisms were used, respectively. The average of the test results was reported. In this research, a self-designed equipment based on the Darcy law was employed for the water permeability test. In this test, the slice specimens of the dimensions of 100 mm × 100 mm × 20 mm, which were cut from the 100-mm cube specimens (see Figure 5), were used to measure the water permeability of SCLC. Prior to the test, four side faces and the top surface of the slice were sealed with epoxy resin. At the centre of the top surface of the slice, a circle area of 50 mm in diameter was uncovered. Then, the slice was placed in an immersion tank that was filled with de-aired water until the SCLC slice was fully saturated. The permeability coefficient (*K*) is expressed as velocity. The *K* is defined as, under a hydraulic gradient unit, the average flow velocity that happens through the entire area of the cross-section of a sample. Figure 6 is the schematic drawing of the equipment that was used in the permeability test.

## 3. Results and Discussion

### 3.1. Properties of Fresh SCLC

#### 3.1.1. Slump Flow Test

##### Effect of the Sand Ratio of MS and LS

**Figure 7:**
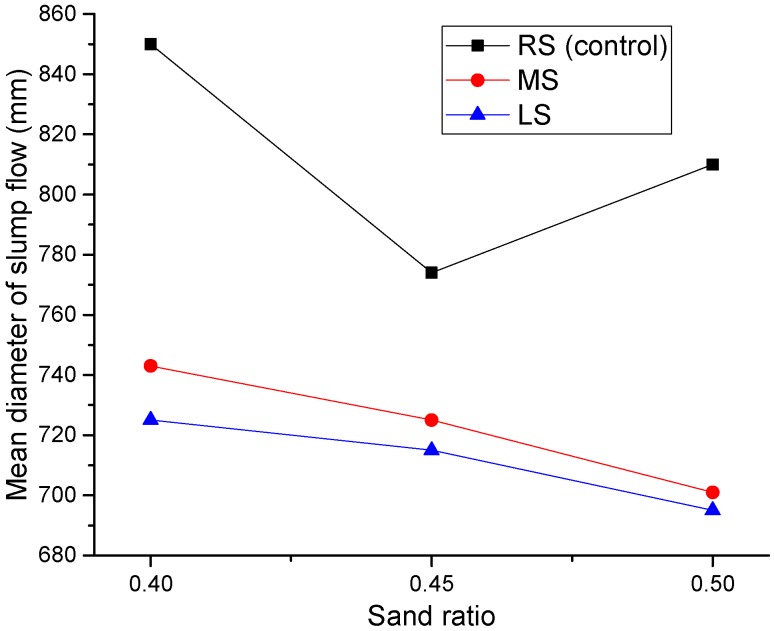
Effect of the MS and LS sand ratio on the SCLC slump flow.

Figure 7 shows the effect of the MS and LS sand ratios on the mean slump flow diameter of the SCLC. In general, the slump flow decreases with the increase of the sand ratio for all SCLC mixes, including the control SCLC (RS-SCLC). The highest values of slump flow were observed when the sand ratio for both MS and LS was 0.4. When the sand ratio was increased to 0.45, the slump flows of RS-SCLC, MS-SCLC and LS-SCLC were reduced by 3%, 9% and 1%, respectively. The increase of fine aggregate greatly increases the total surface area of the aggregates, and the cement paste per unit surface area of aggregate available for providing lubrication is reduced; and thus, the flowability of the concrete is reduced [28]. When the sand ratio was increased to 0.50, the slump values of SCLCs made with MS and LS were further reduced, and the reductions were around 5% and 3%, respectively. In contrast, RS-SCLC recovered a part of its slump loss, and the slump flow reduction was lowered to 4% only when the sand ratio was 0.5. The slump recovery is probably due to the ball bearing effect as the volume of spherical fine aggregate in the mix increased. Based on the slump flow results, the sand ratio of 0.4 is recommended for SCLC with either MS or LS.

**Figure 8:**
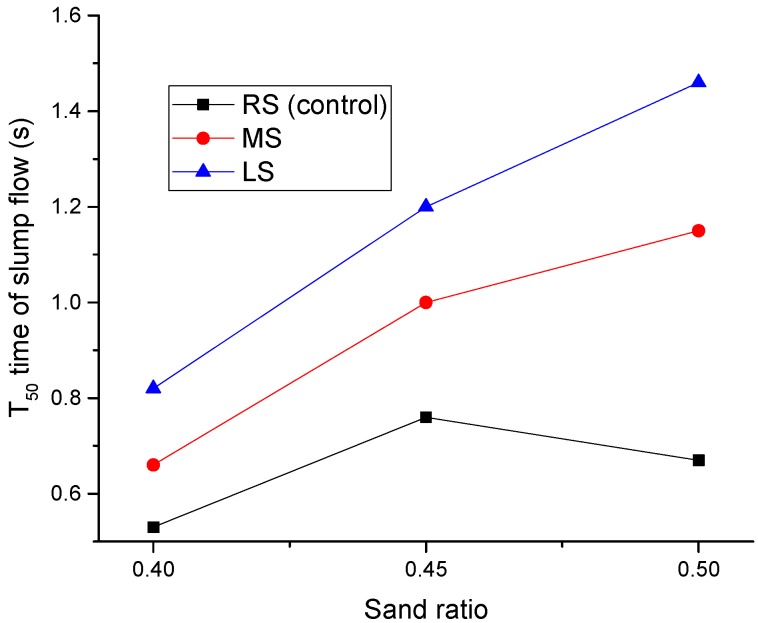
Effect of the MS and LS sand ratio of SCLC on the T_50_ time of slump flow.

Figure 8 shows the effect of the sand ratio on T_50_ times. For SCLCs made with MS and LS, their T_50_ times increased consistently with the increase of the sand ratio. As mentioned earlier, the particle shapes of MS and LS are angular, and thus, the increase in the sand ratio would greatly increase the total surface area of the aggregate, thus consequently reducing the flowability of SCLC. For RS-SCLC, the sand ratio effect on T_50_ is similar to that on slump flow. Based on the slump flow results, it can be identified that the flowability of the SCC made with angular fine aggregates can be reduced when the sand ratio is higher than 0.4.

##### Effect of Fine Aggregate Types

**Figure 9:**
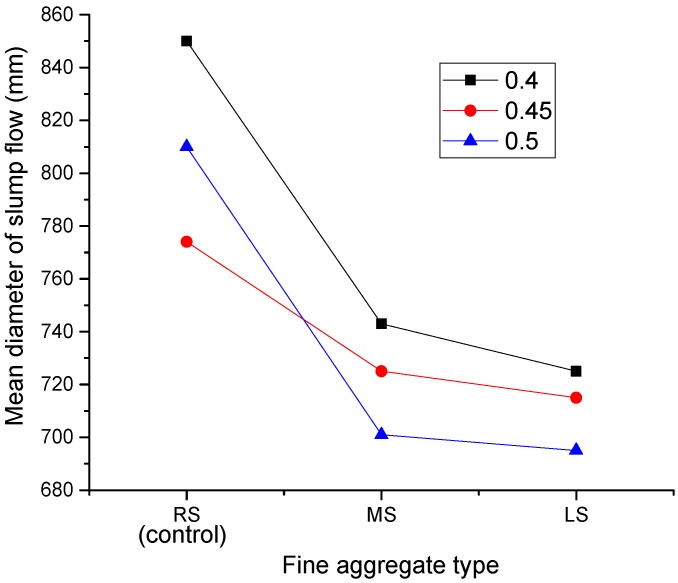
Effect of fine aggregate types (MS and LS) on SCLC slump flow.

Different aggregates may have different shapes and grading, and it is well known that the shape and grading of fine aggregates greatly influence concrete workability. Figure 9 shows the effect of MS, LS and RS (control) fine aggregates on slump flow results. It can be observed from the figure that, regardless of the sand ratio, RS-SCLC showed the highest slump flow values (between 775 mm and 850 mm), whereas LS-SCLC showed the lowest results (between 695 mm and 730 mm). There are two reasons for the comparatively lower fluidity observed for LS-SCLC. First, the particle shape of LS is angular, which results in an increase of the friction between the particles, as well as the amount of cement paste required. Second, LS is lighter in weight than RS. The research [29,30,31] shows that the aggregate density and packing density can influence the rheological properties of mortar or concrete. These reasons can be utilized to explain the experimental phenomena of similar research about the effect of particle shape on the flowability of fresh cement-based mixtures.

Despite the grading and fineness modulus of MS being much different compared to those of LS, the slump flow values of MS-SCLC are similar to those of LS-SCLC at all sand ratios, as shown in Figure 9. In contrast, the slump flow values of MS-SCLC and RS-SCLC are significantly different, though the grading and fineness modulus of MS and RS are similar. It seems that the effect of the particle shape of fine aggregate on SCLC’s flowability is greater than the effect of its grading and fineness modulus.

**Figure 10:**
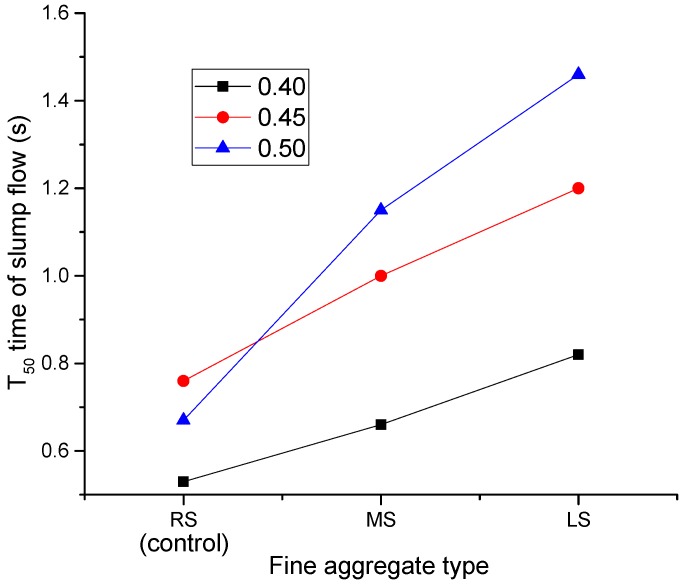
Effect of fine aggregate types (MS and LS) on the T_50_ time of slump flow.

Figure 10 shows the effect of fine aggregate types on T_50_ times. RS-SCLC (control) showed the lowest T_50_ times compared to the MS-SCLC and LS-SCLC mixes, and the difference became significant when the sand ratio increased from 0.4–0.45. The reason is mainly due to the ball bearing effect contributed by the spherical RS aggregate in the mix. LS-SCLC showed the highest T_50_ times at all sand ratios. The reasons are mainly due to the angular shape of LS and its relatively low fineness modulus. All SCLC mixes showed the greatest T_50_ times at a sand ratio of 0.5. When the sand ratio increases, the influence of fine aggregate on SCLC viscosity becomes more and more notable.

#### 3.1.2. L-Box Test

##### Effect of Sand Ratio

**Figure 11:**
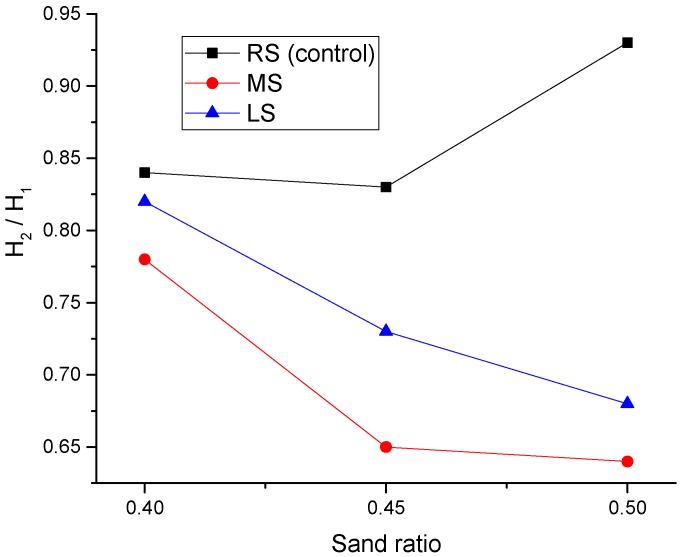
Effect of the MS and LS sand ratio of SCLC on H_2_/H_1_.

Figure 11 displays the effect of the sand ratio on the H_2_/H_1_ blocking ratio, and the trend is very similar to that observed for slump flow values. As stated in the previous section, the L-box test measures the SCLC passing ability, thus quantifying the suitability of the SCC for use in a member with congested reinforcement. From the figure, it is clear to see that the sand ratio has a significant impact on the blocking ratios, especially for MS-SCLC and LS-SCLC. It can be noticed that the ratios of H_2_/H_1_ generally declined with the increase of the sand ratio. When the sand ratio increased to 0.45, the H_2_/H_1_ ratios of RS-SCLC, MS-SCLC and LS-SCLC were reduced by 1%, 16% and 12%, respectively. At a 0.5 sand ratio, the reductions for MS-SCLC and LS-SCLS were both 18%. The reasons are similar to what has been discussed in the previous section for the slump flow test results. However, it is worthy to note that there is a dramatic increase in the blocking ratio of RS-SCLC at a sand ratio of 0.5, which is even higher than its value at a 0.4 sand ratio by 11%. Due to the particle shape of both MS and LS being angular, and that of RS being spherical, therefore, it seems that the ball bearing effect of RS particles plays a more significant role in the blocking ratio than the slump flow.

**Figure 12:**
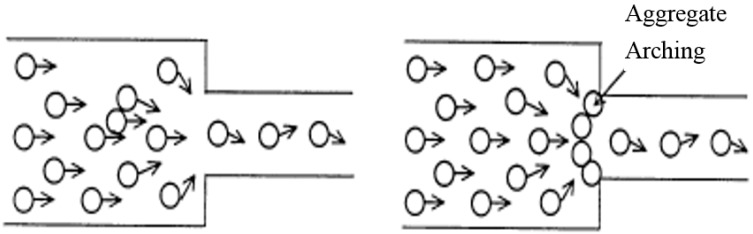
Mechanism of the blocking of SCLC.

From Figure 11, it can be shown that the passing ability of RS-SCLC can be improved with the increase of the sand ratio. Figure 12 shows a two-dimensional illustrative blocking mechanism of coarse aggregates. From the figure, it can be known that the aggregate arching can be reduced by increasing the cement paste volume among the SCLC mixtures. The increased sand ratio means that the volume percentage of fine aggregate in the total aggregate volume is increased; therefore, the increased sand ratio will lead to an increase of the paste volume. As a result, for RS-SCLC, the increase of the sand ratio would improve its passing ability.

##### Effect of Fine Aggregate Type

**Figure 13:**
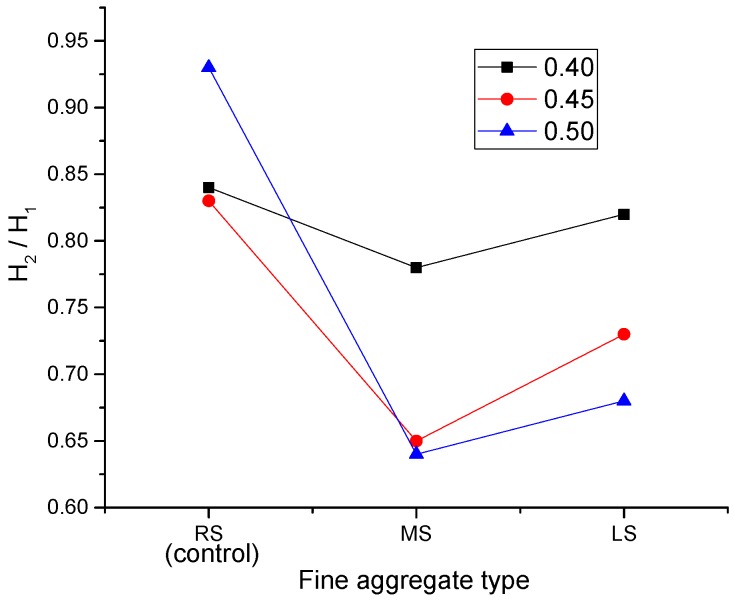
Effect of fine aggregate types (MS and LS) on H_2_/H_1_.

Figure 13 shows the effect of different fine aggregates on the H_2_/H_1_ ratio. Likewise, the trend is similar to that observed for slump flow. It can be clearly shown that RS-SCLC gave the best passing ability compared to other SCLC mixes studied. MS-SCLC showed the lowest results at all sand ratios. Comparing to the particle size and grading of the three kinds of fine aggregates in Table 2, it can be known that the particle size of MS is greater than those of RS and LS. The percentage of MS particles with a 2.36–5 mm size is as high as 37.3%. In contrast, the percentage for RS is only 9.3%. For LS, more than a 90% particle size was located in the range of 0.6–1.18 mm. Therefore, the effect of fine aggregate type on SCLC passing ability mainly relies on the particle size and grading of fine aggregate.

### 3.2. SCLC Compressive Strength

#### 3.2.1. Effect of Sand Ratio

**Figure 14:**
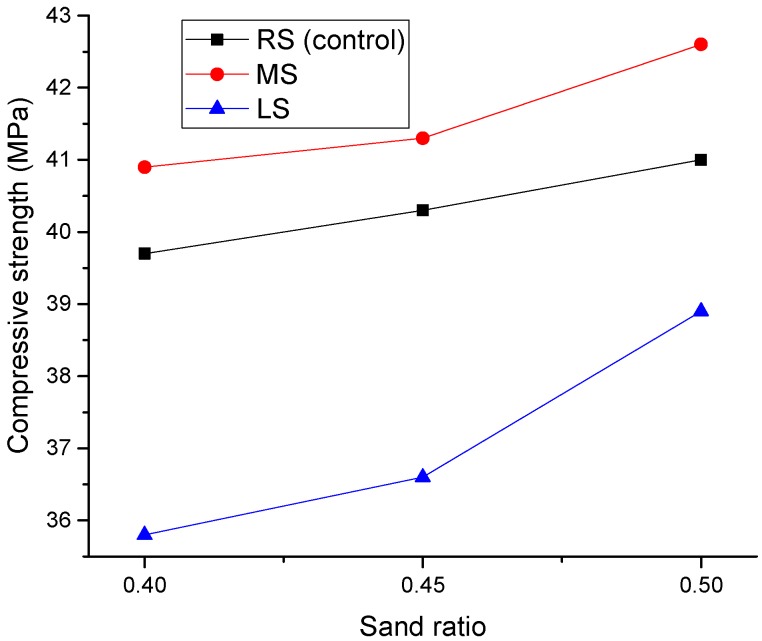
Effect of the MS and LS sand ratio on compressive strength.

Figure 14 displays the effect of the sand ratio on the compressive strength of SCLCs. It can be seen that the compressive strength of SCLC increases with the increase of the sand ratio, and the significance became obvious when the sand ratio was increased to 0.5. When the sand ratio was 0.5, the compressive strength values of RS-SCLC, MS-SCLC and LS-SCLC were increased by 6%, 3% and 8%, respectively. Similar findings on self-compacting normal concrete with different sand ratios were reported by other research [32,33]. Based on the present results, it seems that the effect of sand ratio on the compressive strength of SCLC is similar to that on ordinary SCC.

#### 3.2.2. Effect of Fine Aggregate Type

**Figure 15:**
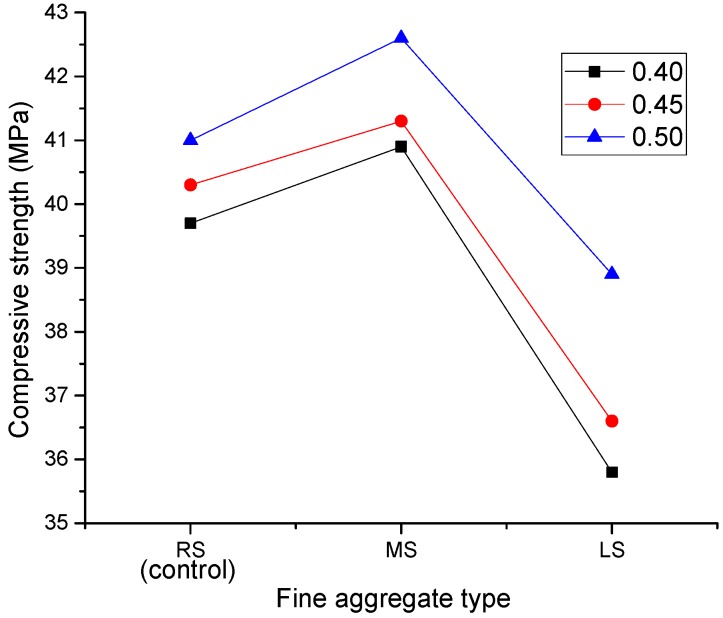
Effect of fine aggregate types (MS and LS) on compressive strength.

Figure 15 displays the effect of different fine aggregates on the compressive strengths of SCLCs. From the curve, it can be clearly shown that MS-SCLC presented the highest compressive strength, while LS-SCLC presented the lowest strength. The reason for the higher compressive strength of MS-SCLC is probably due to the presence of fine limestone powder in MS, which could accelerate the cement hydration and lead to an increase of the concrete strength. Previous research [20,34,35] also showed similar observations.

**Figure 16:**
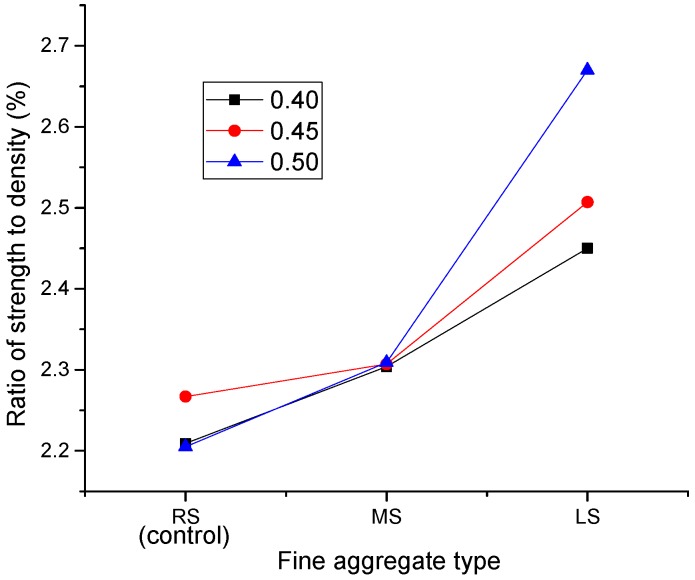
Effect of fine aggregate types (MS and LS) on the ratio of strength to density of SCLC.

Among the fine aggregates studied, LS is a lightweight and low strength fine aggregate. It is believed that LS is a weak component in the matrix of SCLC; therefore, LS-SCLC showed the lowest strength. Lightweight concrete can be seen as a two-phase material comprised of LWA and a mortar matrix. The lower strength of LS can influence the mortar strength, and then, the mortar strength can influence the concrete strength. Figure 16 displays the effect of different fine aggregates on the ratio of strength (MPa) to density (kg/m^3^) of SCLC. From Figure 16, it can be shown that, the ratio of strength to density of LS-SCLC is the highest and higher than others by 15%, though its compressive strength is the lowest among the SCLC mixes and lower than those of other two SCLCs by 8%. It is also worthy to note that the strength to density ratio of SCLC mixes increased with an increase of the sand ratio, in which LS-SCLC showed the highest strength at a sand ratio of 0.5. The results displayed that LS-SCLC has a great potential for the application in buildings for its high strength/density ratio.

### 3.3. Static Elastic Modulus

**Figure 17:**
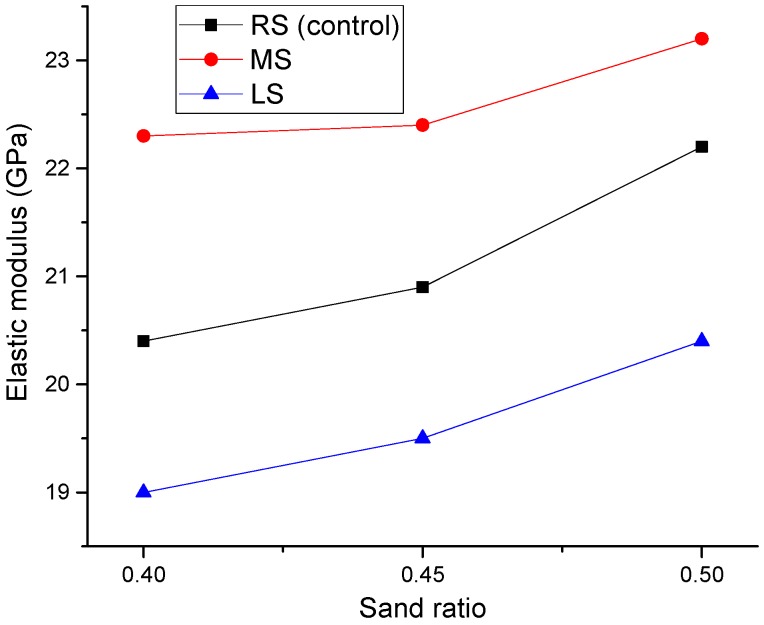
Effect of the MS and LS sand ratio of SCLC on the elastic modulus.

Figure 17 shows the effect of MS and LS sand ratios on the elastic modulus of SCLC. Similar to the compressive strength results, the elastic moduli of SCLCs also increased with the increase of the sand ratio. From Figure 14 and Figure 17, it can be seen that the increased sand ratio can lead to an increase of the mechanical properties of SCLC.

**Figure 18:**
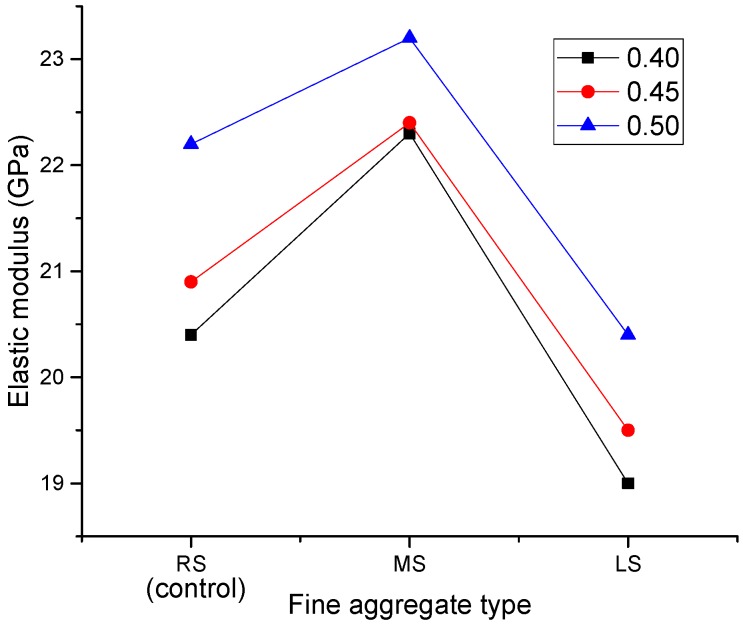
Effect of fine aggregate types (MS and LS) on the elastic modulus.

Figure 18 shows the effect of fine aggregates on the elastic modulus of SCLCs. Likewise, the trend is very similar to that observed for the compressive strength results. From the curve, it can be seen that MS-SCLC showed the highest elastic modulus, whereas LS-SCLC showed the lowest elastic modulus. Among the SCLC mixes studied, MS-SCLC showed the best mechanical properties, while LS-SCLC gave the worst mechanical properties.

### 3.4. Water Permeability Coefficient

**Figure 19:**
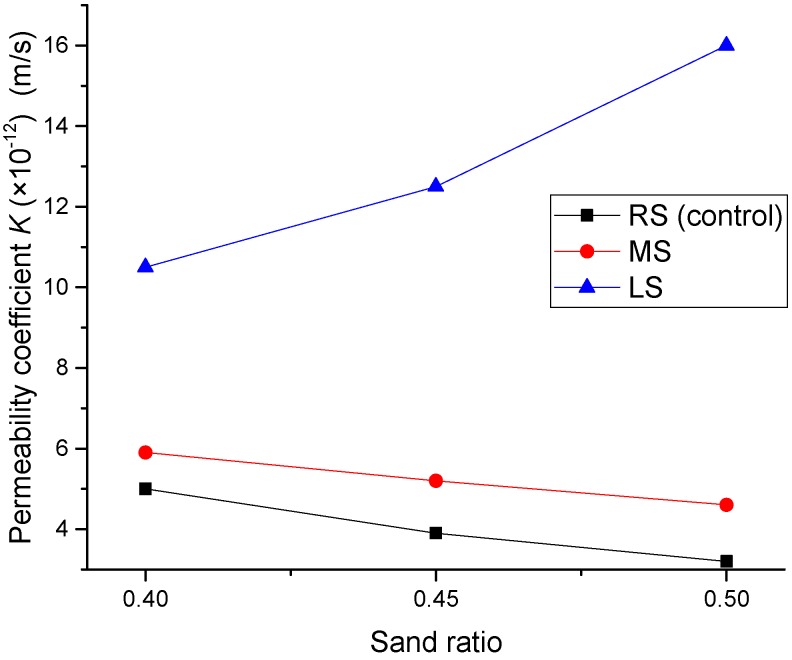
Effect of the MS and LS sand ratio on the permeability coefficient.

Figure 19 presents the effect of MS and LS sand ratios on the permeability coefficient of the SCLCs. For both MS-SCLC and RS-SCLC, their resistance to water permeability increased with the increase of the sand ratio. At a sand ratio of 0.5, the permeability coefficient of MS-SCLC and RS-SCLC was reduced by 18% and 36%, respectively. It is known that, in a certain range of sand ratios, the void content of concrete under good compaction tends to decrease with the increase of normal fine aggregate content, and thus, the permeability can be reduced. However, LS-SCLC showed the opposite results. Its permeability coefficient increased with the sand ratio, as shown in Figure 19. When the sand ratio was increased from 0.4–0.5, the permeability coefficient of LS-SCLC was increased by nearly 50%. The reason for this phenomenon might be due to the porous nature of LS fine particles.

**Figure 20:**
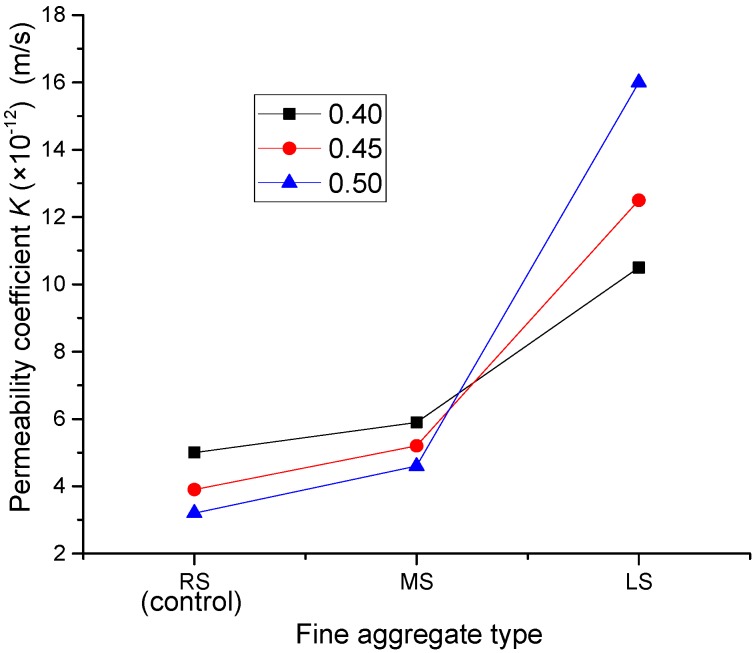
Effect of fine aggregate types (MS and LS) on the permeability coefficient.

Figure 20 shows the influence of fine aggregates of MS and LS on the permeability coefficient of all SCLC mixes. It can also show that LS-SCLC gave the highest permeability coefficient or least resistance to water penetration. The control SCLC (RS-SCLC) gave the best resistance to water permeability. However, the differences between MS-SCLC and the control SCLC are not remarkable. In contrast, the water permeability coefficients of LS-SCLC are greater than those of control SCLC under different sand ratios. The possible reason for the high permeability of LS-SCLC is mainly due to the porous nature of LS fine aggregate, and so, the resistance to water penetration is the least among other SCLC mixes studied.

## 4. Conclusions

Based on the above test results and discussions, the following conclusions can be drawn.

(1) Compared with the workability of control concrete (RS-SCLC), T_50_ times and slump flow results indicated that higher MS and LS sand ratios can reduce the flowing ability of self-compacting concrete. Based on the test results, a sand ratio of 0.40 is recommended for MS-SCLC and LC-SCLC.
(2) Despite the grading and fineness modulus of MS being much different compared to those of LS, the slump flow values of MS-SCLC are similar to those of LS-SCLC at all sand ratios. In contrast, the slump flow values of MS-SCLC and RS-SCLC are significantly different, though the grading and fineness modulus of MS and RS are similar. Therefore, it can be concluded that the effect of particle shape of fine aggregate on SCLC’s flowability is greater than the effect of its grading and fineness modulus.
(3) Compared with the mechanical properties of RS-SCLC, MS-SCLC gave the highest compressive strength and elastic modulus, whereas LS-SCLC showed the lowest strength and elastic modulus. Nevertheless, the ratio of strength to density of LS-SCLC is the highest, and the ratio increased remarkably with the increase of the sand ratio.
(4) Compared with the water permeability coefficient of RS-SCLC, both MS-SCLC and LS-SCCL showed less resistance to water penetration than RS-SCLC. However, the difference in permeability results between MS-SCLC and RS-SCLC is within 20% and is considered acceptable. LS-SCLC showed the highest permeability coefficient, and its permeability coefficient increased with the increase of the LS sand ratio.

## Figures and Tables

**Table 1:** Properties of lightweight aggregate (LWA).

Properties	Value
Bulk density (kg/m^3^)	670
Particle density (kg/m^3^)	1175
Water absorption (1 h) wt %	9.2
Tube crushing strength (MPa)	6.6
Grading (wt %)	
>10 mm	1.53
5~10 mm	85.50
2.36~5 mm	9.25
<2.36 mm	3.72

**Table 2:** Properties of the fine aggregates.

Type	Particle Density (kg/m^3^)	Fineness Modulus	Grading (wt %)				
			2.36~5 mm	1.18~2.36 mm	0.6~1.18 mm	0.3~0.6 mm	<0.3 mm
RS	2600	2.77	9.3	22.6	20.3	31.0	16.7
MS	2600	3.45	37.3	20.12	13.27	9.35	19.96
LS	1727	2.96	–	2.6	91.0	5.98	0.42

**Table 3:** Mix proportion of the SCLCs per cubic meter.

SCLC Group	No.	Cement/kg	PFA/kg	Water/kg	Fine Aggregate/kg	LWA (Pre-Wetted)/kg	Superplasticizer (Litre)	Viscosity Agent (Litre)	Sand Ratio
Control group	C-RS-1	330	220	220	592	401	5.5	0.75	0.40
	C-RS-2	330	220	220	665	368	5.5	0.75	0.45
	C-RS-3	330	220	220	739	334	5.5	0.75	0.50
Studied groups	C-MS-1	330	220	220	592	401	5.5	0.75	0.40
	C-MS-2	330	220	220	665	368	5.5	0.75	0.45
	C-MS-3	330	220	220	739	334	5.5	0.75	0.50
	C-LS-1	330	220	220	393	401	5.5	0.75	0.40
	C-LS-2	330	220	220	442	368	5.5	0.75	0.45
	C-LS-3	330	220	220	491	334	5.5	0.75	0.50

Note: For all mixtures, cement + pulverized fuel ash (PFA): 550 kg/m^3^; water/binder ratio = 0.40; PFA content: 40 wt %.

## Abbreviations

The following abbreviations are used in this manuscript:

MS	manufactured sand
LS	lightweight sand
RS	river sand
LWA	lightweight aggregate
SCC	self-compacting concrete
SCLC	self-compacting lightweight concrete
RS-SCLC	SCLC made with river sand
MS-SCLC	SCLC made with manufactured sand
LS-SCLC	SCLC made with lightweight sand
